# Blink Detection Using 3D Convolutional Neural Architectures and Analysis of Accumulated Frame Predictions

**DOI:** 10.3390/jimaging11010027

**Published:** 2025-01-19

**Authors:** George Nousias, Konstantinos K. Delibasis, Georgios Labiris

**Affiliations:** 1 Department of Computer Science and Biomedical Informatics, University of Thessaly, 35131 Lamia, Greece; gnousias@uth.gr; 2 Department of Ophthalmology, General University Hospital of Alexandroupolis, 68131 Alexandroupolis, Greece; glampiri@med.duth.gr

**Keywords:** blink detection, 3D CNN, 3D autoencoder, 3D ResNet, prediction accumulator, signal analysis

## Abstract

Blink detection is considered a useful indicator both for clinical conditions and drowsiness state. In this work, we propose and compare deep learning architectures for the task of detecting blinks in video frame sequences. The first step is the training and application of an eye detector that extracts the eye regions from each video frame. The cropped eye regions are organized as three-dimensional (3D) input with the third dimension spanning time of 300 ms. Two different 3D convolutional neural networks are utilized (a simple 3D CNN and 3D ResNet), as well as a 3D autoencoder combined with a classifier coupled to the latent space. Finally, we propose the usage of a frame prediction accumulator combined with morphological processing and watershed segmentation to detect blinks and determine their start and stop frame in previously unseen videos. The proposed framework was trained on ten (9) different participants and tested on five (8) different ones, with a total of 162,400 frames and 1172 blinks for each eye. The start and end frame of each blink in the dataset has been annotate by specialized ophthalmologist. Quantitative comparison with state-of-the-art blink detection methodologies provide favorable results for the proposed neural architectures coupled with the prediction accumulator, with the 3D ResNet being the best as well as the fastest performer.

## 1. Introduction

Blink detection and extraction of blinking patterns is considered an essential component in applications like human–computer interaction (HCI) [1] systems, sentiment computing, monitoring fatigue and alertness [2,3], or behavioral studies [4]. Blinking patterns can also serve as important non-invasive biomarkers for several pathologies. More specifically, altered blink rates can be indicators of neurological or ocular conditions, as well as for a series of ophthalmological and systemic diseases or even with more severe pathologies or mental diseases [5,6], such as Parkinson’s disease [7,8], Tourette syndrom [9], hemifacial spasm [10], dry eye syndrome [11] or even schizophrenia [12], making blink recognition a valuable diagnostic tool. In the transportation and industrial sectors [13], reduced or irregular blink patterns can help to identify fatigue or drowsiness, consequently preventing accidents and enhancing safety. Moreover, in assistive communication systems [14], blink recognition serves as a non-invasive, intuitive way of interaction for individuals with severe physical limitations. Therefore, the automatic detection and analysis of blinks is a crucial and versatile step in many artificial intelligence pipelines.

Using image processing and machine learning techniques, as well as sensor-based tracking, blink detection systems capture eyelid motion with high precision. Many of these methods utilize facial landmarks [15], using deep neural networks like convolutional neural networks (CNNs) or vision transformers. Blink detection systems can improve user’s experience, ensuring safety, and enable accessibility in diverse contexts.

Thus, automatic blink detection and identification of blinking patterns can bridge critical gaps between human needs and the capabilities of machines, offering solutions for more accurate, on-time diagnosis and improving the interaction between human and machines.

In this work, we propose a complete blink detection framework based on video sequences of the participants’ upper face, comparing various architectures of neural networks combined with signal segmentation techniques applied to a prediction accumulator for each video frame. The main contributions of this work are

- The training and application of an object detector (YOLOX [16]) that extracts the regions around each eye from each frame of a video sequence, since the zoom factor of the images renders other methods of eye-detection not applicable (i.e., Mediapipe [17]).
- Embedding temporal information in three-dimensional (3D) input into convolutional neural networks (3D CNN, 3D autoencoder and 3D ResNet), with the third dimension spanning a typical blink duration, combined with a simple classifier in order to classify the image sequences during training.
- The proposed inference mode (blink detection in unseen videos) that utilizes one accumulator for each eye, aggregating predictions during a single forward pass of all $N_{s}-$subsequences in the unseen video with step = 1. The accumulators are subsequently processed using morphology and watershed segmentation in order to robustly detect blinks of any duration.
- A novel and accurate definition of blink detection metrics that considers many-to-one predicted and actual blinks (and vice versa).

The proposed methodologies were validated and tested in our clinical data and in one public dataset in comparison with other state-of-the-art methods.

## 2. Materials and Methods

### 2.1. Related Work

An early algorithm, which efficiently detects faces and isolates the eye regions was based on Adaboost method and utilized image features based on the integral image [18], has been an initial component in many blink detection methods, especially when acquired images have full-frontal faces. In [19], multi-scale and orientation Gabor filtering were utilized for blink detection, reporting precision 84.62%. In addition, a method using SVM classifier with Haar wavelets and HOG (Histogram of Oriented Gradient) features [20] achieved blink detection accuracy 92.5% and 86% when tested on standard databases and under real world conditions, respectively. A similar method proposed in [21], where Haar-like features were utilized for face detection, reported 95% blink detection accuracy for good illumination conditions and 77% for poor illumination conditions.

In the study of Choi et al. [22], an AdaBoost classifier was proposed as blink detector, achieving 96% accuracy on their own dataset. Additionally, Al-gawwam and Benaissa [23] proposed a facial landmark estimator in order to calculate the vertical distance between the upper and lower eyelids and used Savitzky–Golay (SG) filter to detect blinks with 96.65% precision on standard datasets.

Drutarovsky and Fogelton proposed an eye blink detection algorithm based on the motion vectors of each eye region [24]. A simple finite state machine was used to change the state of each eye and thus detect blinks. Following this approach, in [25], Fogelton and Benesova estimated motion vectors [26] for each pixel of the eye region, extracting the vertical component of the average motion vector and the statistical standard deviation. Those two parameters were fed to a finite state machine that classifies whether the frame can be considered blink or not. A basic constraint in this method is that the head should not be rotating significantly, and it also requires the cropping of the eye.

In [27], Fogelton and Benesova expanded their previous work, utilized the detected motion vectors of the eye region combined with two LSTMs (each 64 units) that accept normal and reversed time input implementing a bidirectional LSTM. The input subsequence length was set to 40 frames and care was taken to extract the subsequences with the blink of a closed eye in the middle of the frames. However, no details were provided on how an unseen video sequence is temporally segmented into 40-frame subsequences. This method also requires the cropping of the eye region, which was performed manually in the reported results.

In a more recent method [28], a Siamese CNN neural network was used as a 256-length feature extractor. These features were fed to a bidirectional LSTM with 256 cells, followed by a dropout layer and a fully connected layer, which performs many-to-many sequence predictions. The training of the Siamese network was performed independently from the LSTM. Although the authors noted a subsequence length of 64 frames, no details were provided on how a given video was split into subsequences, nor how the exact blink start and stop frame were determined. Cropping of eye regions is a prerequisite for this method as well.

In [29], a deep learning encoder–decoder (DLED) architecture, DeepLabv3+ [30], was used, and two different instances of this network were trained to segment the iris and the palpebral fissure regions from each frame independently. In a post-processing step, the distance between the eyelids and the iris diameter was calculated. The estimated fraction of the palpebral fissure height and the moving median iris diameter were used to classify blinks using adaptive thresholds to handle cases of varying patient–camera distance or the patient’s orientation. This is not an end-to-end method; thus, it may be susceptible to erroneous segmentations. In contrast, the DLEDs are trained using individual frames rather than subsequences.

### 2.2. Overview of the Proposed Methodology

**Figure 1:**
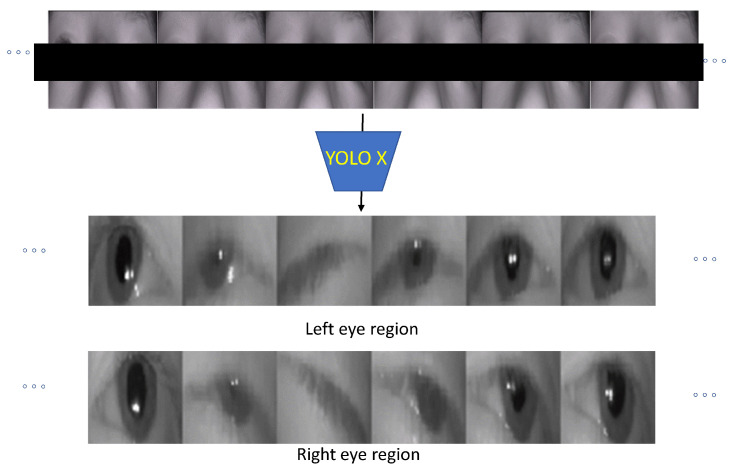
Typical initial video frames and the cropping of the right and left image area 48×48 using the trained YOLOX.

The proposed blink detection method is established through the following basic algorithmic steps. Initially, each eye region (left and right) is identified and detected for each participant using a trained YOLOX [16] neural network detector. The detector predicts the four values of the parameters (upper left corner -x and y coordinate-, width and height) that are used in order to define the bounding boxes that isolate the two eye regions. Typical results of the application of YOLOX are shown in Figure 1.

Following eye detection, the eye region is extracted using YOLOX from 12 consecutive frames (or 300 ms) into a 3D image of size 48×48×12, and it is input into a blink classifier. Three different deep neural architectures were tested as a blink classifier: a 3D ResNet neural network, a simpler 3D CNN encoder and a 3D CNN autoencoder (3D AE). Moreover, there were tested with a few consecutive fully connected layers coupled with a classification layer from the latent space in order to classify the whole subsequence as blink or no blink.

During inference, the 3D eye region extraction is performed on all tuples of 12 consecutive frames with an overlap of 11 frames (equivalently step = 1 frame) for the total video duration. Each 3D eye region is input in the trained classifier, and each one of the 12 frames is characterized as 0 or 1 no blink or blink, respectively. An accumulator *A* is defined for each eye with a non-negative integer value for each frame of the whole video sequence that sums the predicted value for each frame. Considering the accumulator as a 1D discrete signal, the blinks are identified as the peaks of *A*, using the watershed segmentation algorithm.

### 2.3. Eye Region Detection and Preparation of Dataset for the Training Phase

A web-camera setup was used to acquire videos from 17 different subjects during a clinical examination. In order to extract the necessary eye region that was used as input to the classifiers, a YOLOX eye detector was trained. Using the pre-trained neural network for palpebral fissure segmentation [29], the coordinates of the bounding box from 48,000 images from 7 different individuals can be calculated and can be used to train the detector. The YOLOX differs from the classic YOLO methods as it adopts an anchor-free manner and performs other advanced detection techniques like the decoupled head, achieving state-of-the-art results. Having the two bounding boxes (one for each eye, see Figure 1) for each frame and knowing the blink starting and ending points (annotation of ground truth), the dataset can be formed as follows.

**Figure 2:**
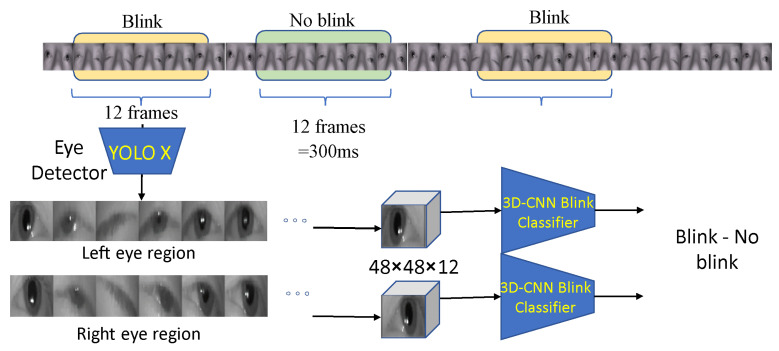
Training data preparation overview. Given the ground truth, starting and ending frames, blink and no-blink $N_{s}$-tuples are formed and used as input for the classifiers.

For all participants, the median blink length/duration was found to be 300 ms or 12 frames correspondingly, considering that videos are captured with frequency 25 frames per second (fps); however, the number of frames where the palpebral fissure is substantially small (i.e., the eye appears closed). It is 85 to 100 ms or 3 to 4 frames at 25 fps. Using the ground truth, we extract a subsequence of length $N_{s}=12$ frames, starting from the annotated beginning of the blinks. Non-blink sequences with a length of $N_{s}=12$ frames are also selected to represent the “No blink” class. In both cases, YOLOX-generated bounding boxes are used to crop the subsequences, which obtain the size of 48×48×12. A graphical overview of the data preparation procedure is shown in Figure 2.

### 2.4. Deep Learning Architectures for Blink Detection

Three different architectures of deep learning neural networks are proposed and tested as blink classifiers. Since blinking is a temporal process of finite duration, we utilized 3D CNNs as proposed architectures, using the cropped subsequences $48\times 48\times N_{s}$ as input. Spatial cropping was generated by YOLOX, as described in the previous subsection. Except for the 3D CNN, we also experimented with an end-to-end two-phase model consisting of a 3D CNN subsequence classifier and of a 3D autoencoder, with a linear combination of the corresponding loss functions. This technique has been shown to be beneficial in many machine learning tasks [31].

After the inference process, the classification results of each classifier are stored in the accumulators and processed, as shown in the next subsection.

#### 2.4.1. Three-Dimensional CNN Architectures for Subsequence Classification

The first tested classifier is the 3D ResNet classifier. It consists of a 50-layer ResNet with 3D convolutions [32], with one (1) stack of four (4) residual blocks, where each convolutional layer of the stack has 64 filters. This architecture was selected since it was already pre-trained on the generic ImageNet dataset, enabling it to successfully capture image characteristics.

**Figure 3:**
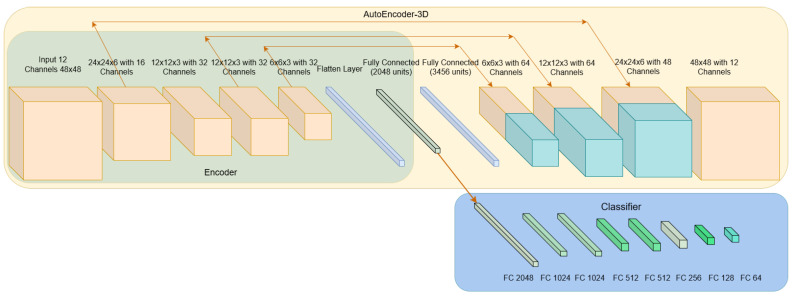
The architecture of the autoencoder with the classifier. Each parallel pipe is the output of the previous block (and the input to the next one).

Furthermore, in order to assess the required complexity of the CNN architecture, a much simpler 3D CNN is also tested as a sequence classifier. This particular architecture “simple 3D CNN” consists of two components, one 3D CNN encoder for feature extraction and one classifier, which is created from repeated blocks of fully connected, ReLU and dropout layers. A graphical representation of this architecture is depicted in Figure 3, where the green box is highlighting the part of the 3D CNN feature extractor and with the blue box being highlighted as the classifier.

#### 2.4.2. Three-Dimensional CNN Autoencoder

Finally, a more complex neural architecture was used as a classifier, a 3D CNN autoencoder along with a classifier. This particular architecture is trying to isolate features in an unsupervised way, aimed at reconstructing the 3D sequence. Alternatively, using descriptive features to classify the subsequence into one of the two classes in a supervised manner can only be carried out when we are given ground-truth labels.

The encoder part of the autoencoder and the classifier (fully connected layers) are identical with the feature extractor and the classifier of the simple 3D CNN, respectively. The encoder part consists of four blocks; each block consists of a 3D convolutional layer, a ReLU activation and a dropout layer. The convolutional layer of the first block, “Conv_1”, with stride [2, 2, 2] and 16 filters with 5 width across the three dimensions, expects the input to be of size 48 × 48 × 12 × 1, resulting in a lower feature map of size 24 × 24 × 6 × 16. The following block, “Conv_2” with stride [2, 2, 2] and 32 filters of size [5, 5, 5], also outputs a lower feature map of size 12 × 12 × 3 × 32. The third convolutional block, “Conv_3”, preserves the same dimensions as the previous “Conv_2” by setting the stride to [1, 1, 1] and retains the same number of filters. The last convolution block, “Conv_4”, reduces the size of the first three dimensions into 6 × 6 × 3 × 32, due to stride [2, 2, 1] and preserving the same number of filters. Finally, the output of “Conv_4” block is flattened and fed to a fully connected layer, which defines the latent space, with 2048 units.

From each one of the “Conv_1”, “Conv_2” and “Conv_4” blocks, a skip connection is created, and the outputs of those blocks are connected and concatenated with the input of the corresponding 3D transposed convolution layer of the decoder’s three blocks.

The decoder part consists of three blocks of repeated 3D transposed convolutional and ReLU layers, while the last 3D convolutional layer followed by a Clipped-ReLU with ceiling 255 are the last layers that will give the predicted/reconstructed output. An example of this architecture is shown in Figure 3, where the size of each output feature map is available along with the skip connections. The skip connections are used in order to minimize the information loss due to the compression and decompression of an encoder and decoder, respectively. Also, their usage will help the autoencoder and the reconstruction loss function to quickly converge in the solution. The autoencoder learns to reconstruct the given input using the characteristics of latent space, while the classifier is utilizing them in order to classify the input sequence as blink or no-blink.

### 2.5. Supervised Training Mode

Considering the blink detection problem as a classification task, the objective is to minimize the error between the predicted probability distribution and true distribution, namely the cross-entropy. Due to the fact that the problem is binary (“Blink”, “No Blink”), the binary cross-entropy function is selected. It is defined in Equation (1). This loss function is selected for the two out of three architectures, the simple 3D CNN and the 3D ResNet classifiers. Let us denote by $t_{j}$ the given ground-truth label for *j* subsequence, with $p_{j}$ being the predicted probability of *j* subsequence belonging to the same class and *N* being the number of total frames. The training loss function is defined as

(1) $$L_{cross}=-\frac{1}{N-N_{s}}\left[\sum_{j=1}^{N-N_{s}}t_{j}log\left(p_{j}\right)+\left(1-t_{j}\right)log\left(1-p_{j}\right)\right]$$

While the above classifiers are exclusively based on the cross-entropy loss, the loss function of the 3D CNN autoencoder is a combination of two loss functions, the cross-entropy and the mean squared error $L_{rec}$ between the predicted (reconstructed) image and the input image, as follows:

(2) $$L_{rec}=\frac{1}{N-N_{s}}\sum_{i=1}^{N-N_{s}}{\left(Y_{i}-{\hat{Y}}_{i}\right)}^{2}$$

where $Y_{i}$ and ${\hat{Y}}_{i}$ are the 48 × 48 × 12 input image and the reconstructed output of the decoder, respectively. $L_{rec}$ calculates how well the decoder can reconstruct the input image sequence based on the features of the latent space. Consequently, the compressed features in the latent space are expected to be quite descriptive. Finally, the total loss for the 3D autoencoder is defined in the following equation as a weighted combination of Equations (1) and (2). The weights were used so that the network considered the classification task as being more significant than the task of reconstruction. It was noticed during experimentation that equal weights result in $L_{rec}$ converging quicker than $L_{cross}$, leading to an overfitted autoencoder and an underfitted classifier during the training phase.

(3) $$L_{total}=0.9\times L_{cross}+0.1\times L_{rec}$$

### 2.6. Inference Mode: Identifying Blinks in Unseen Videos

A common issue in time sequence analysis, such as video, is the segmentation of the sequence into subsequences and their individual classification.

Many of the referenced works on blink detection do not adequately clarify the process of inference in the case of video sequences. Segmenting a previously unseen video sequence into constant-length subsequences will inevitably split a blink event into two subsequences. Thus, inference in these cases may be incorrect. The proposed method consists of interference subsequences of constant length $N_{s}$ with step equal to 1. Thus, each frame will be “seen” by the deep learning model $N_{s}$ times. Specifically, we may assume the number of frames with a palpebral fissure as substantially small (i.e., the eye appears closed). i.e., for them to be M=3 or 4 frames (equivalently 85 to 100 ms at 25 fps). The significant frames of each blink will be split into two adjacent subsequences *M* times, whereas the blink significant frames will be contained into a single subsequence $N_{s}-M$ times. In our application, we selected the subsequence length of $N_{s}=12$ frames or an equivalent time of 300 ms. Setting $N_{s}=12$ ensured that, on average, each frame was expected to be correctly classified 8 to 9 time. Otherwise, it could have been wrongly classified 3 to 4 times. Therefore, accumulating the predictions for each frame and subsequent accumulator peak detection and segmentation is expected to be immune to the blink split issue.

It is evident that increasing $N_{s}$ would cause a small improvement in the achieved classification metrics; however, it would increase the third dimension of the input to the deep learning architectures, thus increasing the memory requirements and computational burden, with it being significant for both training and testing.

For any unseen video *V* of *N* frames, the following steps are applied: The YOLOX eye detector is applied to generate two cropped sequences around each one of the eyes: ${\hat{V}}_{R},{\hat{V}}_{L}=YOLOX\left(V\right)$, where ${\hat{V}}_{R},{\hat{V}}_{L}\in R^{48\times 48\times N}$. For both ${\hat{V}}_{R},{\hat{V}}_{L}$, the subsequences of length $N_{s}$ and overlap $N_{s}-1$ are fed to the blink classifier *C*, and the prediction for the $N_{s}$-tuple frames is assigned to each frame. The blink classifier *C* may be a simple 3D CNN, a 3D autoencoder or a 3D ResNet, as described in the previous subsection.

The first step is to create an accumulator array *A* that holds the sum of predictions for each frame. For each i=1,…,N, the subsequence $S_{i}=\left(F_{i},F_{i+1},\ldots ,F_{i+11}\right)$ is extracted. The blink classifier *C* is applied to predict the class of the subsequence (0: no blink, 1: blink). The index *i* is increased by 1, and the next subsequence is extracted and fed into the classifier. Thus, each frame *F* is being “seen” by the classifier $N_{s}=12$ times, with an equal number of predictions. An accumulator array *A* of size *N* adds all the predictions for each frame. The algorithmic steps for generating the accumulator for the right and left eye are given in detail in Algorithm 1.
**Algorithm 1** Prediction Accumulator**Input:** video sequence**Output:** the accumulator *A* N← num. of frames in video The YOLOX eye detector is applied to generate two cropped sequences around each of the eyes: ${\hat{V}}_{R},{\hat{V}}_{L}=YOLOX\left(V\right)$, where ${\hat{V}}_{R},{\hat{V}}_{L}\in R^{48\times 48\times N}$ Initialize the accumulator $A_{i}\leftarrow 0,i=1,\cdots N$ **for** k=1 to $N-N_{s}$ **do**      the subsequence of frames $S_{i}=\left(F_{i},F_{i+1},\cdots ,F_{i+11}\right)$ is extracted from ${\hat{V}}_{R},{\hat{V}}_{L}$      The blink classifier *C* is applied to predict the class of the subsequence: $p_{k}=C\left(S_{i}\right)\in \left[0,1\right]$ (0: no blink, 1: blink)      **for** each $i=1,\cdots N_{s}$ **do**           The predicted class is assigned to all frames in the class and the accumulator sums the predictions for each frame $A_{i}=A_{i}+p_{k}$     **end for**  **end for**

**Figure 4:**
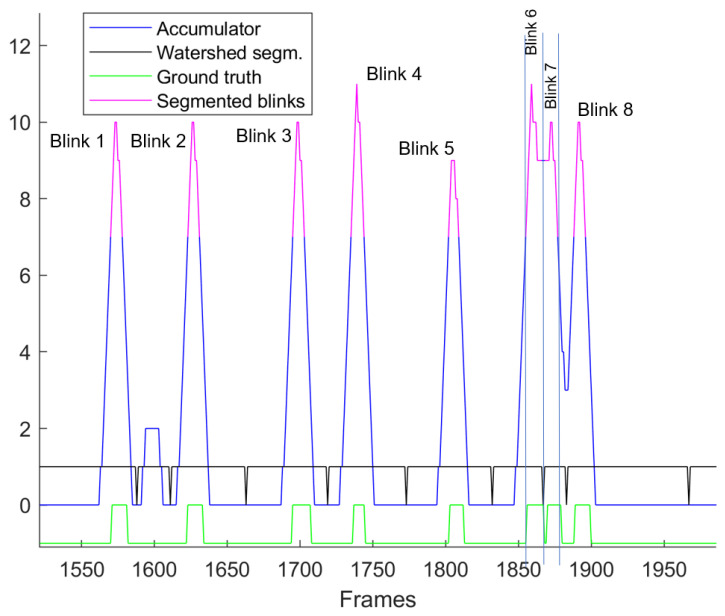
A simplified example of 8 consecutive blinks, where the values of the accumulator, the watershed segments and the ground truth along with the predicted blinks are depicted. It should be noticed that Blinks 6 and 7 are clearly separated due to the watershed algorithm.

It is easily confirmed that $A_{i}$ has integer values in $[0,N_{s}].$ A part of *A* that contains 8 blinks is shown as blue and as a partially red curve in Figure 4. A blink event defined in a frame range will cause the accumulator to exhibit high values within the same range, with the peak value being close to the middle of the blink interval. In order to segment different blinks, the following method is applied. First, the morphological operation of closing is applied to the accumulator with a structuring element $S_{e}$ of length = 3,

$$A=erode(dilate\left(A,S_{e}\right),S_{e}),$$

in order to avoid over-segmentation. The watershed algorithm [33] is applied to the negative of the accumulator, i.e.,

W=watershed(−A).

**Figure 5:**
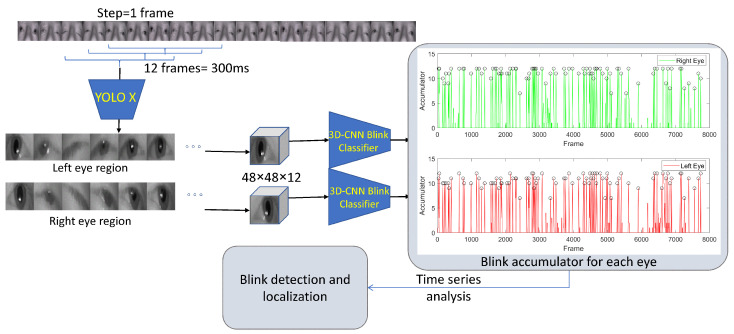
Inference of new video data with a dense overlap (step = 1): 12-tuple eye region frames are fed and classified as blink or no-blink. Blinks are identified utilizing time series analysis and accumulators.

This operation segments the video sequence to consecutive parts, with each one containing one peak of *A* (or one minimum of −A), along with frames not belonging to a blink, as it can be seen by the binary black continuous curve in Figure 4. A threshold *T* is set to each of the watershed segments that indicate the corresponding blinks, which appear as magenta parts of the *A* curve in the same figure. The value of *T* is selected close to half the expected average blink duration $\frac{N_{s}}{2}$. Thus, the start and end frames of each detected blink can be established. For reference, the ground truth, as annotated by ophthalmologists, is shown at the bottom using the thick green curve. In this specific example, Blinks 6, 7 and 8 were successfully identified by the proposed algorithm, despite the fact that they are occurred in rapid succession. The aforementioned process is applied independently to both the right and the left eye of each participant. An overview of the whole process is shown in Figure 5.

### 2.7. Definition of Blink Detection Metrics During Inference

A blink is considered as detected if the intersection over union IOU between the true start–end frames and the predicted ones is not less than 0.2. Considering that there is probability of a single long-duration detected blink to produce IOU≥0.2, we construct the correspondence matrix $R_{ij}$, which marks that the true blink *i* and the predicted blink *j* have $IOU_{ij}\geq 0.2.$ Ideally, $R_{ij}$ should be square, but it is usually not due to imperfect blink predictions. First, let us define the false-negative blinks (FN) as the number of rows in $R_{ij}$ with their sum being equal to 0.

(4) $$FN=\mathrm{number}\;\mathrm{of}\;\mathrm{rows}\;\mathrm{of}\;R\;\mathrm{with}\sum_{j}R_{ij}=0.$$

For the definition of false positives (FPs), let us define the number of excessive predicted blinks corresponding to each one of the true blinks. For example. if three blinks were predicted, all of which correspond to the same true blink, then two of them are considered as FPs. Specifically, the above is expressed as

(5) $$a=\sum_{i:\sum_{j}R_{ij}>1}\left[\sum_{j}\left(R_{ij}\right)-1\right].$$

Similarly, if a column of *R* has a sum of >1, then the number of FP blinks is equal to the sum of the column minus 1. Specifically,

(6) $$b=\sum_{j:\sum_{i}Rij>1}\left[\sum_{i}\left(R_{ij}\right)-1\right].$$

The number of FP (a+b) may count a single predicted blink ij twice as a FP, if and only if the column *j* and the line *i* of the blink both have a sum of greater than 1. Thus, we must subtract these blinks. Specifically,

(7) $$c=\sum_{ij}R_{ij},\;\mathrm{where}\;j:\sum_{i}Rij>1\;\mathrm{and}\;i:\sum_{j}R_{ij}>1.$$

In addition, the number of columns of *R* with a sum equal to 0 is also as FP blinks. Combining all the above, the total number of FP blinks is given by

(8) $$FP=a+b-c+\left(\;\mathrm{number}\;\mathrm{of}\;\mathrm{columns}\;\mathrm{of}\;R\;\mathrm{with}\sum_{j}=0\right)$$

Finally, the number of correctly predicted blinks or true positives (TPs) is defined as

(9) $$TP=\sum_{ij}R_{ij}-FP$$

**Figure 6:**
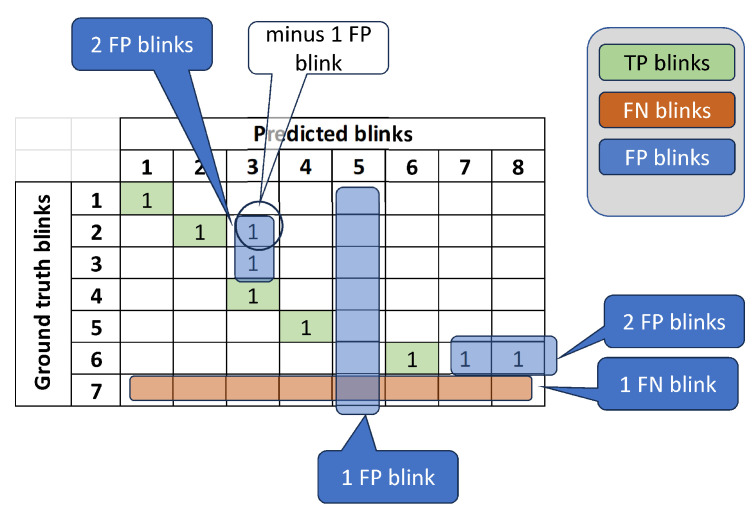
A simplified example of a correspondence matrix between the ground-truth blinks and the predicted ones. TP blinks are indicated by a green color. See text for details.

A simplified example of the corresponding matrix *R* is shown in Figure 6 to demonstrate the relevant definitions. Each row of the matrix represents a ground-truth blink, and each column represents a predicted blink after applying the inference process. Considering Equation (4), the number of false-negative blinks (FNs) is equal to the number of rows of *R* with a sum equal to 0; thus, FN=1 due to the 7th row. The 5th column of *R* is empty; thus, it contributes 1 blink to the FPs. Further, according to Equation (5), a=2 (due to $R_{6,7},R_{6,8}$). Similarly, according to Equation (6), b=2 (due to $R_{2,3},R_{3,3}$). Also, according to Equation (7), c=1 (due to $R_{2,3}$). Thus, FP=4, according to Equation (8). Having calculated the number of false-positive and false-negative blinks, the number of true-positive blinks (indicated by green color) can be readily calculated using Equation (9) as TP=9−4=5.

### 2.8. Clinical Setting

The data were acquired through a prospective study. The protocol that was utilized adhered to the principles of the Declaration of Helsinki, and written informed consent was provided by all participants. The institutional review board of Democritus University of Thrace approved the study protocol (protocol number/date of approval: ES2/Th15/25-2-2021). This clinical study was conducted between October 2020 and March 2021. The official registration number of this study is NCT04828187.

## 3. Results

### 3.1. The Available Dataset

The available dataset consists of 17 patients, 5 of them wearing glasses, each with one video of their frontal face with a duration of between 5 and 10 min. The total number of frames is 162,400, containing 2376 blinks. The ground truth (starting and ending frame for each blink and each eye) was provided by specialized ophthalmologists. The data were split into training and test subsets, using nine and eight patients, respectively. The corresponding number of frames/blinks was 69,400/1204 and 93,000/1172, respectively. All methods under comparison were trained using subsequences from the trained subsets, except for the [29], where deep learning segmentation models were used as already-pretrained models using static images. All methods (including [29]) were validated using the aforementioned test subset. The publicly available dataset “Talking face” [34], provided with the manual annotations of [27], was also used as a separate test subset to assess all methods.

### 3.2. Quantitative Results

Table 1 provides the confusion matrices for each eye separately, as well as the cumulative matrices for both eyes for the three deep learning methodologies and the other competitive methods [27,28,29] under comparison.

Statistics are calculated over the test set. True-negative (TN) blinks are not applicable and are thus indicated by ‘-’. A value of 0.2 was selected for IOU.

The 3D autoencoder, 3D CNN and 3D ResNet achieved more TP blinks than [27,28], with Nousias et al. [29] detecting the maximum number of TPs. Moreover, the three proposed deep neural architectures achieved the least number of false-positive blinks (FPs). Overall, the 3D ResNet achieved the lowest number of FP ans FN blinks when considering all methods, while detecting a very high number of TP blinks.

In Table 2, the classification accuracy and the F1-score are provided for both proposed and state-of-the-art methods under comparison. In terms of F1-score, simple the 3D CNN classifier and 3D autoencoder achieved equivalent classification results (89.72% and 89.63%), surpassing the [29] by ≈2.5% and the [28] by ≈7%. Furthermore, 3D ResNet obtained the highest F1-score (93.25%), with quite promising results. Table 2 also includes the results for the “Talking Face” dataset [34]. The F1-score for the three state-of-the-art methods under comparison was taken from the corresponding publications; however, accuracy was not provided in the publications.

In terms of frame classification, the overall accuracy achieved for our dataset by the methods under comparison was 95.57% for [28], 92.72 for [27], 93.48% for [29], 93.97% for 3D CNN, 93.74% for 3D autoencoder and 94.24% for 3D ResNet.

The required time in seconds for a single-eye forward pass of one 12-frame subsequence, as well as for a single-eye inferencing for a 60 s video, is shown in Table 3. The number of learnable parameters for each proposed model is also available. Please note that the application of YOLOX for eye cropping, which precedes each of the three models, requires 0.08 per frame. The total inference time (YOLOX plus the inferenceof both eyes) for the 60 s video is also given. All measurements were made using the built-in function of MATLAB (version 24.1.0.2689473 (R2024a) Update 6, The Mathworks, Inc., Natick, Massachusetts) on a MS Window 10 computer with the following specifications: i5-9600KF CPU @ 3.70 GHz, 16 GB Ram, GPU: Nvidia GeForce RTX3060 super, 12 GB GPU memory. As it can be observed, 3D ResNet was the fastest model, as well as the best performing one.

## 4. Discussion

Three different deep learning architectures for blink detection in videos acquired under clinical settings were described. A robust approach was proposed for the inference mode of the three architectures for unseen videos. More specifically, all three architectures were applied in an inference mode to cropped video subsequences with a constant length $N_{s}$ and a step set to 1 (overlap equal to $N_{s}-1$). Thus, every cropped subregion of each frame participated in $N_{s}$ input subsequences and therefore received $N_{s}$ predictions. An accumulator added the predictions for every frame, for each eye. A watershed-based segmentation of a negative accumulator segments different blinks and obtains their starting and ending frames.

Table 4 shows the achieved true-positive (TP) and false-positive (FP) blinks for three different values of the IOU threshold. It can be observed that the number of TPs and FPs shows a very slow decreasing trend, as expected, which indicates a low sensitivity of the proposed methods with respect to the threshold.

Quantitative results indicate that all three different deep neural architectures combined with the prediction accumulator achieved better blink detection results than the prior state-of-the-art methods, with the 3D ResNet classifier slightly outperforming the other ones. The usage of the prediction accumulator provides us with the ability to handle video subsequences and to be more robust than seeing subsequences once. The application of a simple threshold and watershed algorithm could be used with any other blink classifier, whether it is a deep learning approach (neural network) or a simple feature-based classifier, like a support vector machine (SVM), allowing for fast signal processing while providing competitive and effective blink detection results.

As already stated, our dataset comprises 17 Caucasian participants from 5 different countries. Five of the participants wore glasses. Under our clinical settings, video acquisition was performed with the participants placed at approximately 10–15 cm from the camera, who were illuminated by two NIR light sources. We plan to substantially increase the number of participants in our future work; however, we do not expect the race distribution to change. Thus, the proposed system is not currently tested for different races nor different camera-to-face distances and orientations. Current experimentation has demonstrated that the proposed system, and especially the eye detection/cropping module, is relatively sensitive to camera-to-face distances. Our future work will also include modifications of the proposed system to classify blinks as complete or incomplete.

## Figures and Tables

**Table 1:** Blink classification results for the test dataset (8 participants, 93,000 frames), with IOU=0.2. Three different confusion matrices are presented, one for each eye and one total, summing blinks of both eyes (ground truth along lines; predictions along columns).

		Total		Right		Left	
		Blink	No Blink	Blink	No Blink	Blink	No Blink
de la Cruz et al. [28]	Blink	952	220	480	106	472	114
	Non Blink	185	-	100	-	85	-
Fogelton et al. [27]	Blink	833	339	479	107	354	232
	Non Blink	68	-	41	-	27	-
Nousias et al. [29]	Blink	1113	59	562	24	551	35
	Non Blink	265	-	155	-	110	-
3D CNN	Blink	1056	116	522	64	534	52
	Non Blink	126	-	64	-	62	-
3D autoencoder	Blink	1050	122	518	68	532	54
	Non Blink	121	-	61	-	60	-
3D ResNet	Blink	1106	66	541	45	565	21
	Non Blink	94	-	40	-	54	-

**Table 2:** The F1-score and accuracy were calculated for the three different proposed classifiers and the previous state-of-the-art methods, using IOU=0.2. The (*) indicates metrics reported in the corresponding publications, while the (-**) indicates metrics not provided in the corresponding publications.

	Our Dataset		Talking Face [34]	
Methods	Accuracy	F1-Score	Accuracy	F1-Score
de la Cruz et al. [28]	70.15	82.46	-**	97.90 *
Fogelton et al. [27]	67.18	80.37	-**	97.10 *
Nousias et al. [29]	77.45	87.29	86.51	92.80
3D CNN	81.36	89.72	90.48	95.00
3D autoencoder	81.21	89.63	91.27	95.44
3D ResNet	87.36	93.25	92.86	96.30

**Table 3:** Number of learnable parameters and required time per eye (s) for inference (forward pass) of one subsequence of 12 frames and 1 min of video for all three proposed models and for a single eye.

Models	12-Frame Sequence Single-Eye Inference	60 s Video, Single-Eye Inference	60 s Total Time for Both Eyes	Learnable Parameters
3D CNN	0.13 s	195 s	510 s	20,590,034
3D autoencoder	0.15 s	225 s	570 s	35,375,507
3D ResNet	0.09 s	135 s	390 s	174,500

**Table 4:** The number of TP and FP blinks for different values of the intersection over union (IOU) threshold during inference mode.

	IOU = 0.2		IOU = 0.3		IOU = 0.4	
Methods	TP	FP	TP	FP	TP	FP
3D CNN	1056	172	1056	162	1050	174
3D autoencoder	1050	166	1052	163	1044	167
3D ResNet	1106	172	1100	166	1102	167

## Data Availability

Training image data can be made available upon request. The pretrained eye region YOLOX detector and the three different neural network classifiers will be made publicly available at the following Github repository: https://github.com/gnousias/blink_detection_accumulator (accessed on 1 December 2024).

## References

[1] Morimoto C.H., Mimica M.R. (2005). Eye gaze tracking techniques for interactive applications. Comput. Vis. Image Underst..

[2] Stern J.A., Boyer D., Schroeder D. (1994). Blink rate: A possible measure of fatigue. Hum. Factors.

[3] Maffei A., Angrilli A. (2018). Spontaneous eye blink rate: An index of dopaminergic component of sustained attention and fatigue. Int. J. Psychophysiol..

[4] VanderWerf F., Brassinga P., Reits D., Aramideh M., Ongerboer de Visser B. (2003). Eyelid movements: Behavioral studies of blinking in humans under different stimulus conditions. J. Neurophysiol..

[5] Cruz A.A., Garcia D.M., Pinto C.T., Cechetti S.P. (2011). Spontaneous eyeblink activity. Ocul. Surf..

[6] Hasan S.A., Baker R.S., Sun W.S., Rouholiman B.R., Chuke J.C., Cowen D.E., Porter J.D. (1997). The role of blink adaptation in the pathophysiology of benign essential blepharospasm. Arch. Ophthalmol..

[7] Kimura N., Watanabe A., Suzuki K., Toyoda H., Hakamata N., Fukuoka H., Washimi Y., Arahata Y., Takeda A., Kondo M. (2017). Measurement of spontaneous blinks in patients with Parkinson’s disease using a new high-speed blink analysis system. J. Neurol. Sci..

[8] Ogawa K., Okazaki M., Mori H., Hidaka T., Tomioka Y., Tanaka K., Uemura N., Akiyama M. (2022). Comparative blink analysis in patients with established facial paralysis using high-speed video analysis. J. Craniofacial Surg..

[9] Cohen D.J., Detlor J., Young J.G., Shaywitz B.A. (1980). Clonidine ameliorates Gilles de la Tourette syndrome. Arch. Gen. Psychiatry.

[10] Osaki M.H., Osaki T.H., Garcia D.M., Osaki T., Gameiro G.R., Belfort R., Cruz A.A.V. (2020). Analysis of blink activity and anomalous eyelid movements in patients with hemifacial spasm. Graefe’s Arch. Clin. Exp. Ophthalmol..

[11] Craig J.P., Nichols K.K., Akpek E.K., Caffery B., Dua H.S., Joo C.K., Liu Z., Nelson J.D., Nichols J.J., Tsubota K. (2017). TFOS DEWS II definition and classification report. Ocul. Surf..

[12] Stevens J.R. (1978). Eye blink and schizophrenia: Psychosis or tardive dyskinesia?. Am. J. Psychiatry.

[13] Dawson D., Searle A.K., Paterson J.L. (2014). Look before you (s) leep: Evaluating the use of fatigue detection technologies within a fatigue risk management system for the road transport industry. Sleep Med. Rev..

[14] Ezzat M., Maged M., Gamal Y., Adel M., Alrahmawy M., El-Metwally S. (2023). Blink-To-Live eye-based communication system for users with speech impairments. Sci. Rep..

[15] Soukupova T., Cech J. Eye blink detection using facial landmarks. Proceedings of the 21st Computer Vision Winter Workshop.

[16] Ge Z. (2021). Yolox: Exceeding yolo series in 2021. arXiv.

[17] Kartynnik Y., Ablavatski A., Grishchenko I., Grundmann M. (2019). Real-time facial surface geometry from monocular video on mobile GPUs. arXiv.

[18] Viola P., Jones M.J. (2004). Robust real-time face detection. Int. J. Comput. Vis..

[19] Li J.W. (2008). Eye blink detection based on multiple Gabor response waves. Proceedings of the 2008 International Conference on Machine Learning and Cybernetics.

[20] Pauly L., Sankar D. (2015). A novel method for eye tracking and blink detection in video frames. Proceedings of the 2015 IEEE International Conference on Computer Graphics, Vision and Information Security (CGVIS).

[21] Królak A., Strumiłło P. (2012). Eye-blink detection system for human–computer interaction. Univers. Access Inf. Soc..

[22] Choi I., Han S., Kim D. (2011). Eye detection and eye blink detection using adaboost learning and grouping. Proceedings of the 2011 20th International Conference on Computer Communications and Networks (ICCCN).

[23] Al-gawwam S., Benaissa M. (2018). Robust eye blink detection based on eye landmarks and Savitzky–Golay filtering. Information.

[24] Drutarovsky T., Fogelton A. (2014). Eye blink detection using variance of motion vectors. Proceedings of the European Conference on Computer Vision.

[25] Fogelton A., Benesova W. (2016). Eye blink detection based on motion vectors analysis. Comput. Vis. Image Underst..

[26] Farnebäck G. (2003). Two-frame motion estimation based on polynomial expansion. Proceedings of the Image Analysis: 13th Scandinavian Conference, SCIA 2003.

[27] Fogelton A., Benesova W. (2018). Eye blink completeness detection. Comput. Vis. Image Underst..

[28] de la Cruz G., Lira M., Luaces O., Remeseiro B. (2022). Eye-lrcn: A long-term recurrent convolutional network for eye blink completeness detection. IEEE Trans. Neural Netw. Learn. Syst..

[29] Nousias G., Panagiotopoulou E.K., Delibasis K., Chaliasou A.M., Tzounakou A.M., Labiris G. (2022). Video-based eye blink identification and classification. IEEE J. Biomed. Health Inform..

[30] Chen L.C., Zhu Y., Papandreou G., Schroff F., Adam H. Encoder-decoder with atrous separable convolution for semantic image segmentation. Proceedings of the European Conference on Computer Vision (ECCV).

[31] Nellas I.A., Tasoulis S.K., Georgakopoulos S.V., Plagianakos V.P. (2023). Two phase cooperative learning for supervised dimensionality reduction. Pattern Recognit..

[32] He K., Zhang X., Ren S., Sun J. Deep Residual Learning for Image Recognition. Proceedings of the IEEE Conference on Computer Vision and Pattern Recognition (CVPR).

[33] Beucher S. (1994). Watershed, hierarchical segmentation and waterfall algorithm. Mathematical Morphology and Its Applications to Image Processing.

[34] Talking Face Video. https://personalpages.manchester.ac.uk/staff/Timothy.F.Cootes/data/talking_face/talking_face.html.

